# Differential Gaze Patterns on Eyes and Mouth During Audiovisual Speech Segmentation

**DOI:** 10.3389/fpsyg.2016.00052

**Published:** 2016-02-02

**Authors:** Laina G. Lusk, Aaron D. Mitchel

**Affiliations:** ^1^Neuroscience Program, Bucknell UniversityLewisburg, PA, USA; ^2^Department of Psychology, Bucknell UniversityLewisburg, PA, USA

**Keywords:** speech segmentation, visual speech, visual prosody, multisensory integration, eye tracking, language acquisition

## Abstract

Speech is inextricably multisensory: both auditory and visual components provide critical information for all aspects of speech processing, including speech segmentation, the visual components of which have been the target of a growing number of studies. In particular, a recent study (Mitchel and Weiss, 2014) established that adults can utilize facial cues (i.e., visual prosody) to identify word boundaries in fluent speech. The current study expanded upon these results, using an eye tracker to identify highly attended facial features of the audiovisual display used in Mitchel and Weiss (2014). Subjects spent the most time watching the eyes and mouth. A significant trend in gaze durations was found with the longest gaze duration on the mouth, followed by the eyes and then the nose. In addition, eye-gaze patterns changed across familiarization as subjects learned the word boundaries, showing decreased attention to the mouth in later blocks while attention on other facial features remained consistent. These findings highlight the importance of the visual component of speech processing and suggest that the mouth may play a critical role in visual speech segmentation.

## Introduction

To comprehend speech, listeners do not simply rely on their ears; cues in the facial gestures of the speaker play a key role in speech perception (Massaro, 1998). These cues, known as *visual speech*, provide important linguistic information, particularly when the corresponding auditory input is degraded or unclear (Sumby and Pollack, 1954; Grant and Seitz, 2000). Visual speech has been shown to enhance a wide variety of speech and language processes, including speech comprehension (Sumby and Pollack, 1954), phoneme categorization (Patterson and Werker, 2003), language discrimination (Soto-Faraco et al., 2007; Weikum et al., 2007; Navarra et al., 2014), and phonetic learning (Teinonen et al., 2008; van der Zande et al., 2014).

Visual speech conveys both segmental visemic (Fisher, 1968) and suprasegmental (e.g., Cvejic et al., 2012) linguistic cues through various features of the face, such as mouth and head movements or eye gaze direction. The utility of these cues and features depends on the specific speech task and the accompanying auditory information. Different tasks, such as identifying words or emotional content from speech, require different information, and thus may differ in the degree to which they rely upon information gleaned from one cue or another (Lansing and McConkie, 1999, 2003). In the present study, we explore the relative contributions of different facial areas in one particular language task: speech segmentation.

Before acquiring a vocabulary, infants must learn to identify where one word ends and another begins (Jusczyk et al., 1999), a process referred to as speech segmentation. Isolating words from continuous speech is a substantial perceptual challenge since there are no consistent gaps between words in normal speech (Saffran, 2003), and words are rarely said in isolation, even to infants when parents are trying to teach their children words (Woodward and Aslin, 1990). In fact, only 5–10% of utterances are words in isolation, increased just to 20% when parents are specifically asked to isolate words when speaking to their infants. Given the crucial role of speech segmentation in developing language, research into the availability and usage of segmentation cues provides insight into the cognitive and perceptual mechanisms that allow humans to learn and comprehend language.

Previous research has focused almost exclusively on identifying the auditory cues facilitating speech segmentation, such as stress patterns (e.g., Jusczyk et al., 1999; Cunillera et al., 2006), pitch/prosody (e.g., Schön et al., 2008; de Diego-Balaguer et al., 2015), coarticulation (e.g., Mattys, 2004), the distribution of speech sounds (e.g., Saffran et al., 1996), or a hierarchical combination of these cues (e.g., Mattys et al., 2005). However, in recent years there has been increased interest in the role of faces in speech segmentation. These studies have largely investigated how faces complement statistical cues to word boundaries (e.g., Sell and Kaschak, 2009; Mitchel and Weiss, 2010) or how temporal synchrony in multimodal stimulation enhances or complements cues available in the speech stream (e.g., Cunillera et al., 2010; Thiessen, 2010; Brar et al., 2013). For example, Hollich et al. (2005) demonstrated that temporally synchronous videos (faces and non-face dynamic stimuli) can enhance the detection of target words in continuous speech when the speech stream has been obscured by noise. Moreover, recent findings indicate that statistical learning mechanisms supporting speech segmentation can operate over audiovisual percepts combining faces with voices (Mitchel et al., 2014), suggesting that these mechanisms are modality interactive (Mitchel and Weiss, 2011; Glicksohn and Cohen, 2013; Mitchel et al., 2014; Frost et al., 2015) rather than modality specific (Conway and Christiansen, 2005, 2006). This growing body of research suggests that learners utilize visual cues, and particularly faces, to enhance speech segmentation. However, an open question is whether faces, independent of the auditory stream, provide cues that would support speech segmentation. Given the substantial acoustic noise present in a standard speech environment (Hollich et al., 2005), visual segmentation cues might provide an important complement to cues carried in the auditory signal.

The only study, to date, to address this question investigated adults’ ability to segment speech using visual prosodic cues (Mitchel and Weiss, 2014). Visual speech displays provide reliable cues to the prosody of the synchronous speech stream (Yehia et al., 2002; Cvejic et al., 2012). Since acoustic prosody signals word boundary locations (Jusczyk et al., 1999), Mitchel and Weiss (2014) proposed that visual prosody may similarly help learners segment speech. To examine the word boundary cues contained within visual speech, Mitchel and Weiss (2014) reduced acoustic and statistical segmentation cues to a minimum, forcing subjects to rely upon visual cues for successful segmentation. The authors paired a video of an actor lip-syncing with an auditory stream of an artificial language, which contained minimal cues to segmentation. When presented in isolation, adults failed to correctly segment the auditory stream. However, following audiovisual familiarization, participants performed above chance on the same audio-only test, suggesting that participants were able to extract visual boundary cues and apply this knowledge to segment the audio stream. Furthermore, segmentation performance with the video was dependent on the actor being aware of the correct word boundaries. If the actor was misinformed about the word boundaries, performance again dropped to chance. Mitchel and Weiss (2014) therefore demonstrated that visual speech conveys cues to the location of word boundaries, and that adults are able to utilize these cues to segment speech.

However, it is still unclear which aspects of visual speech provided the most salient segmentation cues in Mitchel and Weiss (2014). The results of both production (e.g., Yehia et al., 2002) and perception (e.g., Swerts and Krahmer, 2008) studies reveal variability in the facial cues that carry prosodic information (reviewed in Cvejic et al., 2012). Several different facial features have been linked to prosody, including lip aperture (Yehia et al., 2002), rigid head movements (e.g., Munhall et al., 2004; Kitamura et al., 2014), and eyebrow movements (Kim et al., 2014). In addition, across different studies, there is conflicting evidence as to whether the primary visual prosodic cues are in the upper portion of the face (e.g., Swerts and Krahmer, 2008; Cvejic et al., 2010), the lower portion of the face (e.g., Lansing and McConkie, 1999; Yehia et al., 2002), or that cues are equally available in both regions (e.g., Cvejic et al., 2012). Thus, while Mitchel and Weiss (2014) established the role of visual prosody in speech segmentation, a number of different facial features could have provided these prosodic cues.

Eye-tracking research provides insight into the importance of each facial feature across a variety of linguistic tasks, such as identifying words, emotional content, or prosodic patterns. For example, the mouth is viewed more during speech than during silence, is increasingly viewed as auditory noise increases (Vatikiotis-Bateson et al., 1998; Lansing and McConkie, 2003), and appears important for word identification (Thomas and Jordan, 2004). The eyes, on the other hand, are often the first feature viewed, especially when speech does not occur (Lansing and McConkie, 2003), and appear to be more important for emotional or prosodic judgments than for word identification (Lansing and McConkie, 1999; Buchan et al., 2007; Swerts and Krahmer, 2008). The nose may be a compromise between attractions to the eyes and mouth, particularly during noise (Buchan et al., 2007, 2008), and may serve as a good vantage point for taking in all the features of the face (Lansing and McConkie, 1999). Finally, a recent study found that both task demands and familiarity with the language modulate attention to the mouth or eyes during audiovisual speech perception (Barenholtz et al., 2016).

The studies reviewed above indicate that gaze direction during an audiovisual speech task is dependent in part on the specific demands of the task. The various features of the face provide different types of information and speech-related cues, and the viewer’s gaze is directed to the regions providing the best cues for the task at hand. This forms the basis of the Gaze Direction Assumption, which postulates that, typically, viewers of a face will spend the most time looking at the features providing the most useful information (Lansing and McConkie, 2003). Since visual prosody can be conveyed by a number of features, and the demands of speech segmentation are distinct from the production (e.g., Yehia et al., 2002) and matching (e.g., Cvejic et al., 2010) tasks previously used to assess visual prosody, the present study aims to identify which facial features learners utilize during visual speech segmentation.

The present study, therefore, uses an eye tracker to assess where participants look during an audiovisual speech segmentation task, adapting the stimuli and procedure of Mitchel and Weiss (2014). We predict that eye gaze patterns during this difficult segmentation task will provide insight into visual speech segmentation and identify the features that convey cues to word boundaries, as postulated by the Gaze Direction Assumption (Lansing and McConkie, 2003). Specifically, we predict that participants will spend the most time viewing the mouth because of the difficulty of the speech segmentation task and the preference for the mouth when an auditory speech signal is difficult to understand. In addition, we predict that gaze patterns may shift as learning progresses, focusing on the cues most relevant to the task at hand, as is seen in early language learning (Lewkowicz and Hansen-Tift, 2012) and consistent with the view that gaze patterns shift as task demands change (Malcolm et al., 2008).

## Materials and Methods

This study was approved by the Bucknell University IRB. All subjects provided written consent before participating in this study. Methods, including artificial language stimuli and segmentation testing using words and part-words, were developed with reference to studies such as Saffran et al. (1996, 1999).

### Participants

Sixty-eight students from Bucknell University participated in this study for academic credit. Participants were monolingual English speakers with no more than 8 years of language experience in any language other than English. In the audiovisual condition, participants’ data were excluded if they viewed less than 65% of the familiarization stream, as this was judged to reflect a failure to follow instructions and attend to the familiarization stream. A total of nine participants failed to meet this criteria and were excluded from the analysis. Participants were not excluded or differentiated based on their use of corrective lenses. The final number of participants included in the analysis was 59, with 30 (24 females) in the audiovisual condition and 29 (16 females) in the audio-only condition. The age of subjects ranged from 18–21 years old.

### Stimuli

The stimuli consisted of an audiovisual familiarization movie and an audio test. In order to replicate the learning exhibited in previous research, we used the same familiarization and test stimuli as was used in the “new aware” condition (Condition 4) of the previous study (Mitchel and Weiss, 2014).

The audio stimulus was an artificial language consisting of six tri-syllabic (CV.CV.CV) words (*bo.ke.taj, pu.taj.bo, ke.gi.da, da.pu.gi, gi.bo.pu, taj.da.ke*). The words were created by synthesizing natural speech syllables in Praat (Boersma and Weenink, 2011) to remove any acoustic cues to word boundaries, such as stress patterns, and then concatenating the syllables into words. This method of synthesis has been used successfully in several prior segmentation studies (Weiss et al., 2009, 2010). The six words were concatenated into a loop of 18 words (each word occurring three times in a pseudo random order). This loop was then repeated 16 times to create a 4-min audio stream containing 288 words. In the audio-only condition, this stream was played three times for a total familiarization of 12 min.

Each syllable was used in each word position, and the sequence order was constrained to prevent reduplication of syllables and to ensure that within-word transitions did not also occur between words. This resulted in a statistical structure where the transitional probability between syllables within words was 0.33 and the transitional probability at word boundaries was 0.11. This difference in probabilities was a consistent but minimal cue to segmentation that in previous research was not sufficient to support learning (Mitchel and Weiss, 2014).

In the audiovisual condition, the speech stream was paired with a digital video of an actor mouthing the words in time with the audio component. The accompanying video stimulus was the “new aware” video used in Condition 4 of Mitchel and Weiss (2014). During the creation of the video, an actor lip-synced to the 18 word audio loop, which was played from a computer. To increase the accuracy of the lip-syncing, the audio was played at 50% speed and then the video was later sped up to match the audio stream. The actor was given a script that contained the 18 words in the audio stream, with word boundaries in the correct locations. Head movements during lip-syncing were minimized by having the actor maintain contact with a fixed point on the wall behind his head. After recording, the audio portion of this video was removed, and the silent video was then was edited, sped up, looped, and synchronized with the audio stream using Adobe Premiere^®^ software. The final video was composed of 16 repetitions of this 15-s clip that were faded in over 1 s at the beginning and end of the clip to remove jerky head movements resulting from looping the clips. The video was 4 min long and consisted of 288 words. The dimensions for the video were 26 cm × 19 cm (22.92° × 16.75°).

The test stimuli consisted of the six audio words (see above) and six audio part-words (*taj.ke.gi, bo.da.pu, da.gi.bo, gi.taj.da, pu.bo.ke, ke.pu.taj*). Part-words were created by combining the third syllable of one word with the first and second syllables of another word. Thus, although these part-words occurred during familiarization, they did not fall in line with the visual cues to word boundaries. The test was the same for the audio-only and audiovisual conditions.

### Procedure

#### Audio-only Familiarization

In the audio-only condition, we used E-Prime 2.0 software to present the audio stream. Participants were instructed to listen to the audio stream and keep their headphones on their head at all times. These instructions remained on the screen while the speech stream was played. The 4-min block was played three times for a total familiarization of 12 min. Between each block there was a 1-min pause.

#### Audiovisual Familiarization

In the audiovisual condition, we used Tobii Studio^®^ software to present the familiarization movie and collect gaze duration data. The video stimulus was displayed on an integrated 17 inch Tobii T60 eye tracker. The eye tracker recorded data at a rate of 60 Hz, and used both bright and dark pupil tracking. Recording latency was 30–35 ms. The maximum accepted gaze angle was 35°, and on average the gaze angle was about 20°. The freedom of head movement was 44 cm × 22 cm × 30 cm at a distance of 70 cm from the eye tracker. Average distance from the eye tracker was about 65 cm.

Before beginning calibration, subject position was adjusted to a distance of 60–65 cm from the eye tracker, with the height of the chair and eye tracker adjusted to center participants in front of the screen. Using Tobii Studio^®^, the eye tracker was then calibrated for each subject using a 5-point calibration. If fixations were reliably located for each calibration point, the experiment proceeded to the familiarization phase; otherwise, the calibration step was repeated. Following calibration, subjects watched the audiovisual movie in three 4-min blocks, with a 1-min pause in between each block. Subjects were instructed to simply watch the screen for the entire duration of each video and were told that there would be a short test following the video. All participants wore noise-canceling headphones during familiarization and the segmentation test. During the pauses in between blocks, a black screen with the instruction “1-min pause” was presented. The three blocks and two pauses were presented continuously from start to finish for a total familiarization of 12 min.

#### Test Phase

Following familiarization, subjects completed a two-alternative forced-choice (2afc) test, presented using E-Prime software, to determine speech segmentation performance. The test was audio-only, and was identical to the test used in Mitchel and Weiss (2014). During the test, participants heard a word and then a part-word (order was counterbalanced), and then were prompted to select which of the two items was the word. Each word was tested against each part word, resulting in 36 test trials.

### Analysis

**FIGURE 1:**
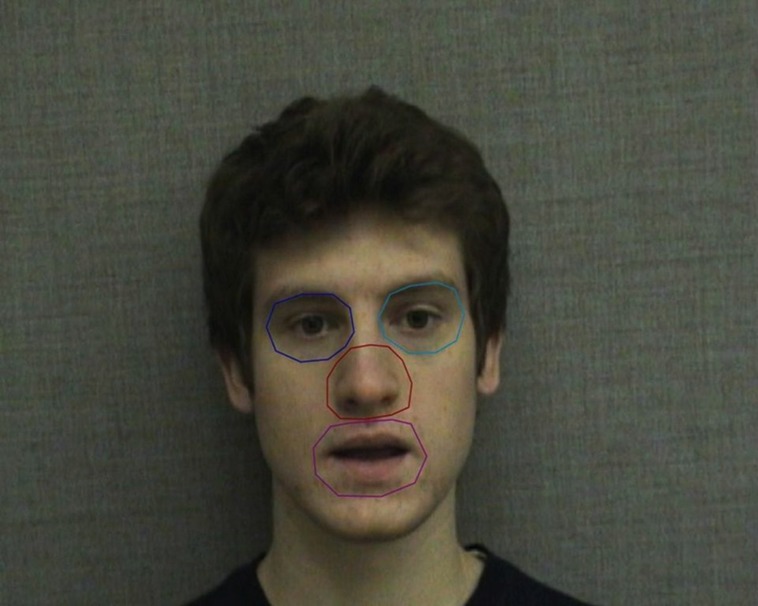
**Areas of interest (AOIs) used in eye gaze analysis**. AOIs correspond to the subjects’ perspective and include the left eye (speaker’s right eye), right eye (speaker’s left eye), nose, and mouth.

Four areas of interest (AOIs) were created for the actor’s face prior to data collection, surrounding the left eye, right eye, mouth, and nose (**Figure 1**). The size and shape of the AOIs as well as the features included were modeled after AOIs in previous studies, particularly Buchan et al. (2008). Each AOI was similar in area and designed to contain the entire feature during speech-related movements (such as lip movement and small head nods). The duration of gazes that fell within these defined regions was used during analysis.

We chose to analyze total gaze duration for two primary reasons. First, familiarization was long and repetitive, and participants tended to have both fewer and longer fixations at the end of familiarization, making fixation count less suitable. Pupillometry and the latency and duration of individual fixations were similarly less suitable due to the long familiarization. Second, pilot data suggested that a shorter familiarization (a single 4-min block) was insufficient for subjects to learn word boundaries; thus, we predicted that segmentation would be dependent in part on viewing time which would be best captured by total gaze duration.

In our analyses, we compared learning in the audio-only and audiovisual trials to confirm that the visual component of the video did in fact convey segmentation cues not present in the auditory component. We also compared overall gaze duration for each defined AOI to identify features that may be contributing to successful segmentation performance. Finally, we compared gaze duration during each block to identify changes in gaze strategy across familiarization.

## Results

### Segmentation Performance

**FIGURE 2:**
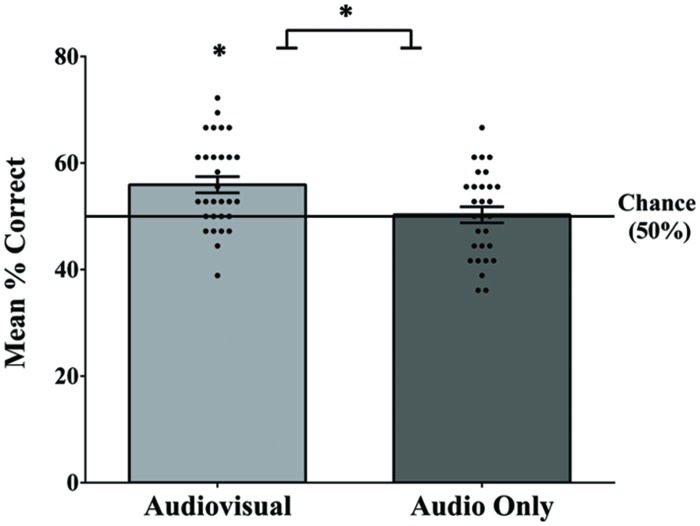
**Mean accuracy, as well as each individual score on segmentation test for the audiovisual and audio-only familiarization conditions**. Performance in the audiovisual condition was significantly above chance and was significantly greater than the audio-only condition. Error bars denote ±1 SEM. ^∗^ denotes significance at 0.05 level.

In order to replicate and verify the earlier findings that visual speech cues enable adults to segment speech (Mitchel and Weiss, 2014), we compared segmentation performance in the visual speech condition to the audio-only baseline condition. The mean number of correct responses in the audiovisual speech condition was 20.13 (*SD* = 2.99) out of 36 (56% accuracy; **Figure 2**). This level of performance, while modest, was significantly above chance (50%), *t*(29) = 3.91, and *p* = 0.001, and was a moderate to large effect size, Cohen’s *d* = 0.71. In contrast, the mean number of correct responses in the audio-only baseline condition was 18.10 (*SD* = 2.93) out of 36 (50%), which was not significantly above chance: *t*(28) = 0.19, *p* = 0.851, and *d* = 0.04. Consistent with our predictions, participants who received the audiovisual familiarization were significantly more accurate on the post-familiarization test than participants who received the audio-only familiarization, as confirmed by an independent samples *t*-test, *t*(57) = 2.63, *p* = 0.011, and *d* = 0.69. Item analyses using repeated measures ANOVAs did not reveal any significant differences in endorsement of individual words in the audio-only [*F*(5,140) = 1.09, *p* = 0.367, and $\eta_{p}^{2}$ = 0.038] or audiovisual [*F*(5,145) = 1.73, *p* = 0.131, and $\eta_{p}^{2}$ = 0.056] conditions.

### Eye-Tracking

**FIGURE 3:**
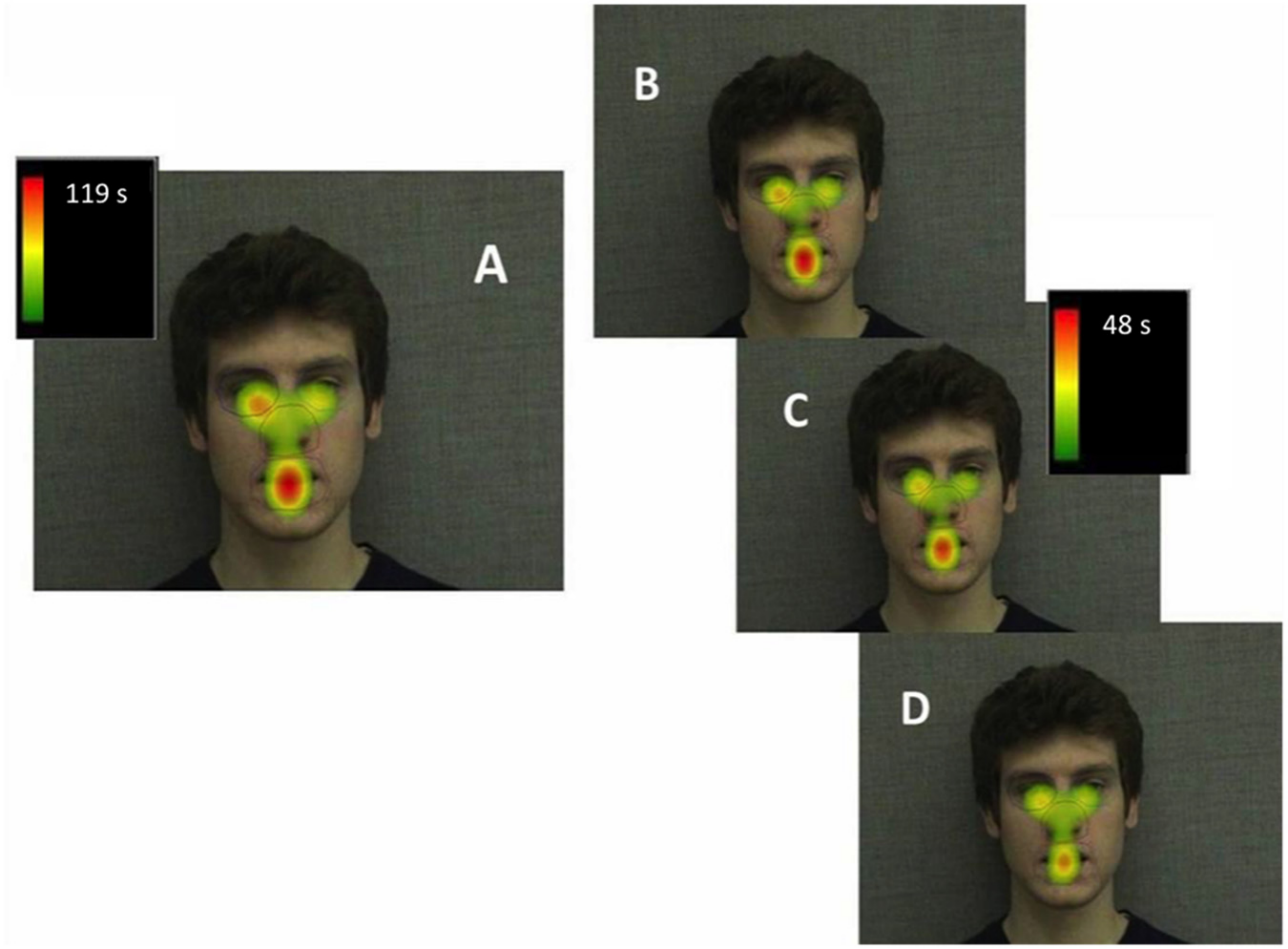
**Heatmap illustrating mean total gaze durations (seconds) in each area of interest across the entire familiarization (A), in block 1 (B), block 2 (C), and block 3 (D)**.

**FIGURE 4:**
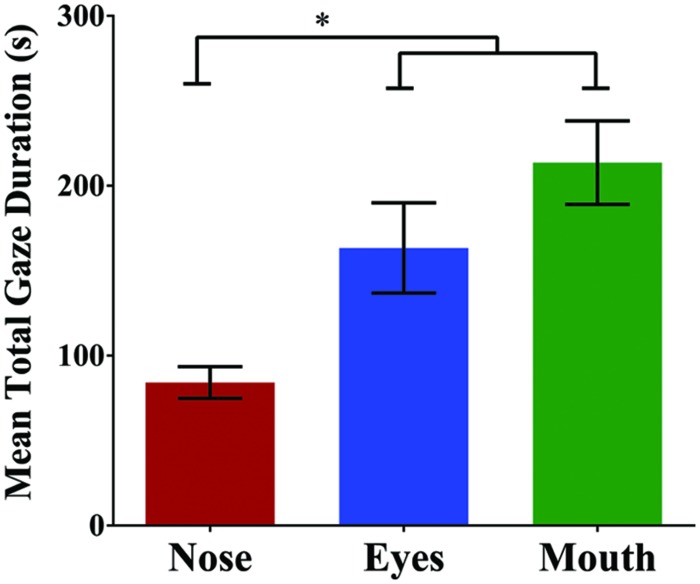
**Mean total gaze duration (seconds) for each of the three facial regions**. Gaze duration on the mouth and eyes was significantly greater than the nose. Error bars denote ±1 SEM. ^∗^ denotes significance at 0.05 level.

To examine participants’ fixation patterns during familiarization, we analyzed total gaze duration in three specific facial regions: the nose, the eyes (left and right summed), and the mouth (**Figure 3**). Although there was a slight viewing bias favoring the actor’s right eye (*M* = 92.71 s) over the left (*M* = 70.63 s), this difference was not statistically significant [*t*(29) = 1.08, *p* = 0.288, and *d* = 0.20]. In addition, previous studies have suggested that such eye gaze biases reflect viewer preferences, rather than speaker asymmetry, and do not have an effect on speech perception or vary with task (Everdell et al., 2007; Guo et al., 2012). For this reason, eye gaze data for each eye was collapsed during analyses. The mean total gaze duration for the nose was 84.20 s (*SD* = 51.02 s), for the eyes was 163.34 s (*SD* = 145.42 s), and for the mouth was 213.58 s (*SD* = 134.54 s; **Figure 4**). A one-way repeated measures ANOVA was performed with Greenhouse-Geisser correction for sphericity due to a significant Mauchly’s test (*p* < 0.001). The ANOVA revealed a significant effect of facial region on gaze durations, *F*(1.25,36.24) = 6.83, *p* = 0.009, and $\eta_{p}^{2}$ = 0.191, with a significant linear trend from nose as the least viewed and mouth as the most viewed [Linear contrast: *F*(1,29) = 25.92, *p* < 0.001, and $\eta_{p}^{2}$ = 0.472]. *Post hoc* pairwise comparisons (Bonferroni correction) confirm that both the mouth and eyes were viewed longer than the nose region (*p* < 0.001 and *p* = 0.039, respectively). Though the mouth had a longer gaze duration than the eyes, this difference was not significant (*p* > 0.05).

**FIGURE 5:**
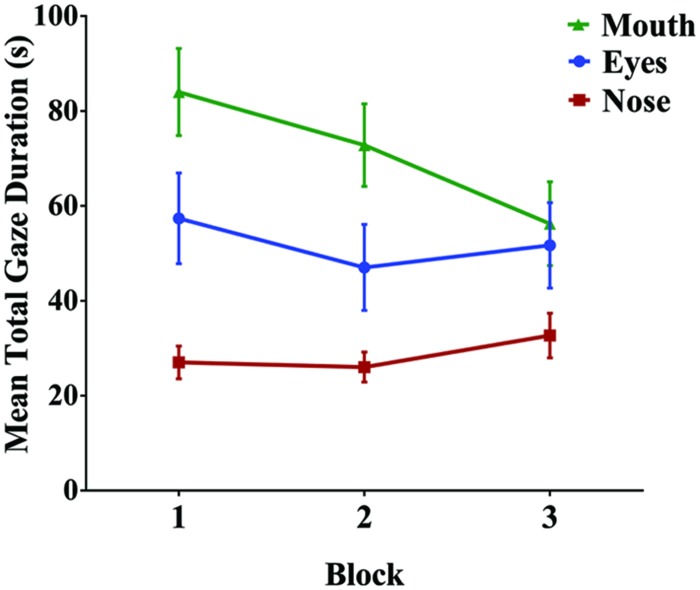
**Mean total gaze duration for each facial region across the three familiarization blocks**. As familiarization progressed, there was a significant decrease in gaze duration on the mouth, but not for the eyes or nose. Error bars denote ±1 SEM.

Familiarization occurred in three blocks; given this, we analyzed changes in gaze patterns across blocks, with the expectation that as participants begin to extract and establish the structure of the language, their gaze patterns may shift to reflect a change in task demands (Malcolm et al., 2008). To assess the effect of block on gaze duration, we conducted a 3 (first, second, and third block) × 3 (nose, eyes, and mouth) repeated measures ANOVA. Once again, region had a significant main effect on gaze duration, *F*(1.27,36.79) = 6.67, *p* = 0.009, and $\eta_{p}^{2}$ = 0.187. In addition, there was a significant main effect of block, *F*(2,58) = 18.08, *p* < 0.001, and $\eta_{p}^{2}$ = 0.384. Finally, there was a significant region by block interaction, suggesting that participants gaze patterns shifted as familiarization progressed (i.e., gaze duration on the mouth decreased across blocks while gaze duration on the eyes and nose remained constant; **Figure 5**): *F*(2.75,79.68) = 4.53, *p* = 0.007, and $\eta_{p}^{2}$ = 0.135. To investigate this interaction further, we conducted three separate one-way repeated measures ANOVAs to test the simple main effect of block on gaze duration for each facial region (nose, eyes, and mouth). For both the nose [*F*(2,58) = 1.81, *p* = 0.174, and $\eta_{p}^{2}$ = 0.059] and the eyes [*F*(2,58) = 2.13, *p* = 0.128, and $\eta_{p}^{2}$ = 0.068], there was no significant difference in gaze duration across blocks. However, there was a significant effect of block on gaze duration in the mouth region [*F*(1.48,42.95) = 9.72, *p* = 0.001, and $\eta_{p}^{2}$ = 0.251]. Bonferroni *post hoc* comparisons confirmed that gaze duration was significantly less in the final block than in the first (*p* = 0.003) or second block (*p* = 0.001), with no significant difference between the first and second block of familiarization (*p* = 0.318).

Not surprisingly, the overall amount of time that participants were attending the facial display decreased across blocks, most likely due to task fatigue [RM ANOVA: *F*(2,58) = 37.15, *p <* 0.001, and $\eta_{p}^{2}$ = 0.562; linear contrast: *F*(1,29) = 51.71, *p <* 0.001, and $\eta_{p}^{2}$ = 0.641]. To ensure that the decline in gaze duration on the mouth across blocks was not simply due to less overall time viewing the face (i.e. time on task), we performed the same block analysis on the relative gaze durations for each region. We normalized viewing time by taking the gaze duration for each region and dividing it by the overall amount of time on task for each block, resulting in a percentage of viewing time spent on a particular region. Since this was percentage data, we used an arcsine transformation to more closely approximate a normal distribution. A 3 × 3 RM ANOVA confirmed a significant main effect of region [*F*(1.25,36.23) = 7.40, *p* = 0.006, and $\eta_{p}^{2}$ = 0.203], no significant overall effect of block [*F*(2,58) = 0.89, *p* = 0.418, and $\eta_{p}^{2}$ = 0.030], and a significant interaction between block and region [*F*(2.86,82.94) = 4.65, *p* = 0.005, and $\eta_{p}^{2}$ = 0.138]. To explore the interaction, we once again conducted three separate one-way RM ANOVAs for each facial region, again as a percentage of time on task. There was a significant effect of block for both nose [*F*(2,58) = 4.01, *p* = 0.023, and $\eta_{p}^{2}$ = 0.122] and mouth [*F*(1.60,46.48) = 6.78, *p* = 0.005, and $\eta_{p}^{2}$ = 0.189], but no significant block effect for eyes [*F*(1.60,46.34) = 1.89, *p* = 0.161, and $\eta_{p}^{2}$ = 0.061]. Linear contrasts indicate that there was a significant *increase* in viewing of eyes [*F*(1,29) = 5.35, *p* = 0.028, and $\eta_{p}^{2}$ = 0.156], whereas there was once again a significant decrease in gaze duration on the mouth across blocks [*F*(1,29) = 6.86, *p* = 0.014, and $\eta_{p}^{2}$ = 0.191]. This analysis therefore confirms the presence of a block effect for the mouth while taking into consideration a decrease in overall viewing time.

**FIGURE 6:**
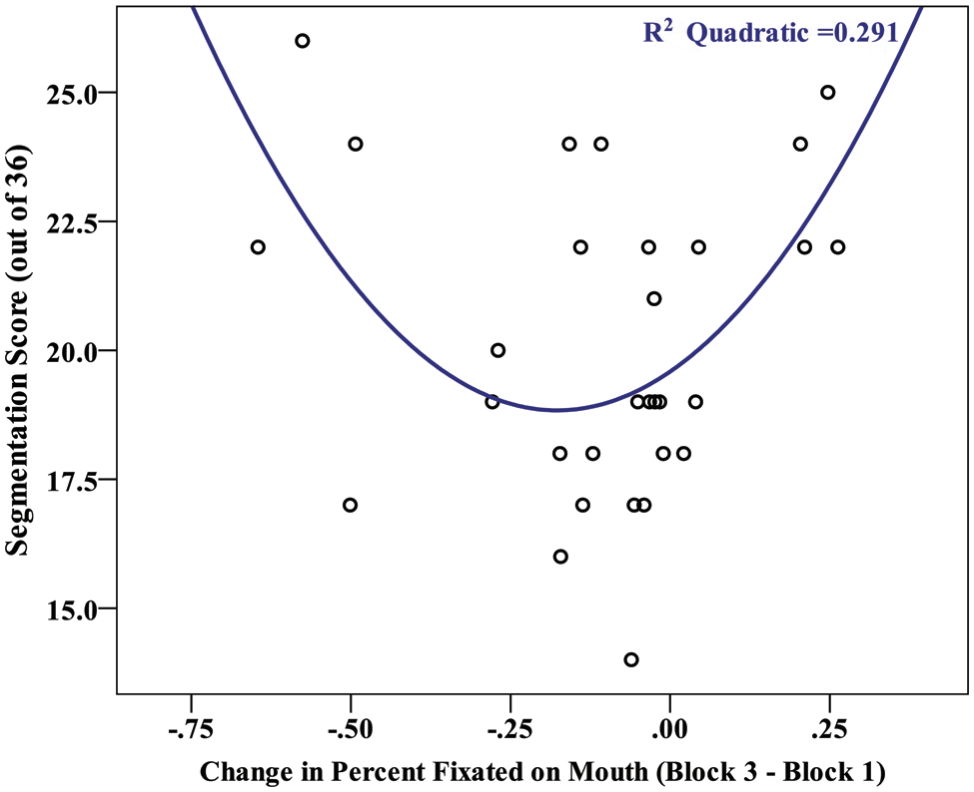
**Scatterplot depicting the relationship between the magnitude of a participants’ gaze shift (Block 3–Block 1 in percent viewing time on the mouth) and their segmentation score (# correct out of 36)**.

Finally, if the decrease in viewing time on the mouth reflects a strategic shift on the part of learners, then we might expect a change in gaze pattern across blocks to be related to overall segmentation performance. To examine this, we estimated the shift in viewing strategy for each participant, using the first familiarization block as a baseline point of comparison. We subtracted the gaze duration for a specific region in the final block from the gaze duration for that region in the first learning block (block 3–block 1). Using this gaze shift metric, we found a significant effect of facial region on the magnitude of gaze shifts, [*F*(2,58) = 4.30, *p* = 0.018, and $\eta_{p}^{2}$ = 0.129], with a *post hoc* contrast revealing that the shift for the mouth (mean change = -10.3%) was significantly different from the eyes and nose (*M* = 3.3%, *M* = 7.0%, respectively). Further examining the gaze shift on the mouth region, a visual inspection of the relationship between gaze shift and segmentation performance (**Figure 6**) suggested that this relationship may be curvilinear. To test this, a polynomial regression was conducted to predict segmentation performance based on gaze shift. There was no significant first order (linear) relationship [*F*(1,28) = 0.16, *p* = 0.899, *R*^2^ = 0.001, and β = -0.024], but there was a significant second order (quadratic) relationship [*F*(2,27) = 5.54, *p* = 0.010, *R*^2^ = 0.291, and β = 0.853). This suggests that individuals with larger gaze shifts on the mouth, irrespective of whether it was an increase or decrease across blocks, exhibited greater levels of learning. We investigated this possibility more directly with a bivariate correlation between segmentation performance and the absolute value of gaze shift for each participant. There was a significant positive correlation between test score and absolute gaze shift, *r*(28) = 0.408, *p* = 0.025, confirming that a larger shift (in either direction) in gaze duration on the mouth is associated with greater learning.

## Discussion

In the current study, we demonstrated that visual speech conveys cues to speech segmentation, replicating findings from Mitchel and Weiss (2014). Participants in the audio-only familiarization condition performed at chance on a test of speech segmentation, while participants in the audiovisual familiarization condition performed significantly above chance and significantly better than participants in the audio-only condition. In addition, eye tracking data during audiovisual familiarization revealed which facial features were most prominent during segmentation. We found significantly higher total gaze duration on the eyes and mouth compared to the nose, with a significant linear trend revealing that participants spent the most time viewing the mouth, followed by the eyes and then nose. Finally, comparison of the three familiarization blocks revealed a block effect in which gaze duration (either absolute or relative) on the mouth significantly decreased in the third block while gaze duration on the eyes and mouth remained nearly constant. Moreover, this shift in gaze duration on the mouth was associated with greater performance on the segmentation task.

These results support our hypothesis that the mouth would be highly attended during familiarization. Previous research has demonstrated that eye gaze patterns are related to task demands (Lansing and McConkie, 1999; Malcolm et al., 2008; Malcolm and Henderson, 2010); thus, the trend toward longer total gaze duration on the mouth in this study suggests that the mouth may be of particular relevance to the task of speech segmentation. Mouth movements are a direct result of articulation, making it a prominent source of visual prosody (Yehia et al., 1998, 2002). In addition, although visual prosody is conveyed in both the upper (Cvejic et al., 2010) and lower regions of the face (Yehia et al., 2002), the realization of these cues is task dependent. The upper half appears to be most pertinent to judgments of sentence or phrase-level prosody (Cvejic et al., 2010). In contrast, the lower half—and the mouth in particular—is most relevant to assessments of word-level prosody, such as lexical stress (Lansing and McConkie, 1999; Yehia et al., 2002). Since auditory segmentation studies have previously identified lexical stress as a cue to word boundary (Jusczyk et al., 1999; Johnson and Jusczyk, 2001), it is likely that visual prosodic cues also operate at the word level; thus, our results are consistent with this dichotomy between facial region and type of prosodic information.

In addition, research with infants has demonstrated the mouth to be highly attended when forming native phoneme categories, with eye gaze shifting back to the default preference for eyes once these categories have been established (Lewkowicz and Hansen-Tift, 2012). This suggests that as learners extract regularities from ambiguous speech input, they pay particular attention to visual cues provided by the mouth. In the current study, the block effect seen for gaze duration on the mouth could similarly be a result of learning. The curvilinear relationship between the magnitude of gaze shift and segmentation performance provides indirect evidence for the possible existence of distinct viewing strategies. Some participants may have focused on the mouth while learning the word boundaries and then shifted their focus to take in other cues, such as social or emotional content. In contrast, a separate subset of participants may have initially fixated on the nose or eyes and then gradually shifted focus to the mouth when their initial strategy did not yield robust representations of word boundaries.

Interestingly, both of these strategies resulted in more accurate segmentation of the speech stream; thus, it may be that learners who adopt a strategy of sampling more broadly from their environment (a common feature of both scenarios described above) are more successful learners. This explanation is consistent with probabilistic (e.g., Bayesian) models of speech segmentation, in which the learner inductively evaluates and updates hypotheses based on the fit between prior expectations and what is observed (Goldwater et al., 2009; see also, Griffiths et al., 2010). In this view, the learner identifies a sample segment of syllables and then updates the conditional probability (i.e., the posterior distribution) that the sample is a word following additional input. The accuracy of this online sampling process has been shown to be impacted by resource limitations, such as memory capacity (Frank et al., 2010), suggesting that the ability to sample more information may facilitate segmentation. It is important to note that since eye-gaze is an online measure and segmentation was measured oﬄine following familiarization, our results only provide indirect evidence of the relationship between gaze behavior and segmentation. Future research may be able to assess both eye-gaze and learning through online measures. For example, tracking eye movements while also recording EEGs, which have been used to assess learning in real time (e.g., Sanders et al., 2002; de Diego Balaguer et al., 2007), would provide direct evidence of whether changes in gaze strategy correspond to learning.

Our results further underscore the importance of the visual component of speech in mechanisms supporting language acquisition. As first demonstrated by Mitchel and Weiss (2014) and replicated here, visual speech provides independent cues to speech segmentation, beyond the cues provided by the auditory speech stream. This builds on research illustrating that visual information enhances learning during auditory speech segmentation (e.g., Hollich et al., 2005; Cunillera et al., 2010; Mitchel and Weiss, 2010; Thiessen, 2010; Mitchel et al., 2014), and highlights the importance of multisensory associations during an early, fundamental component of language acquisition. Similarly, faces play an important role in many other components of language acquisition (e.g., Bertelson et al., 2003; Patterson and Werker, 2003; Weikum et al., 2007; Teinonen et al., 2008; Mani and Schneider, 2013) and there is evidence that adults alter their facial gestures to enhance linguistic cues in visual speech (Green et al., 2010). Consistent with claims that speech may be fundamentally audiovisual (Rosenblum, 2008) and that sensory learning mechanisms, more generally, may have evolved to operate optimally over multisensory input (Shams and Seitz, 2008), the present study suggests that the language learning environment is similarly multimodal. Furthermore, our results support the emerging view that mechanisms for implicitly extracting structure from linguistic input are not fully constrained by sensory modality (Conway and Christiansen, 2005), but are instead prone to cross-modal interactions (Mitchel and Weiss, 2011) and associative integration (Mitchel et al., 2014; see also Frost et al., 2015).

It is important to note that in the present study, we did not gage attention during the passive listening familiarization in the audio-only condition. Thus, unlike in the audiovisual condition, we cannot be certain that participants were actively attending to the audio stream. We consequently cannot preclude the possibility that the greater performance in the audiovisual condition was due to a difference in participants’ attention between conditions. However, previous research suggests that auditory speech segmentation can occur in the absence of focused attention (i.e., passive listening, see Toro et al., 2005) and even in the presence of a low attentional load cover task (e.g., Saffran et al., 1997). It is only when the distracting cover task constitutes a high attentional load that segmentation is impeded (Toro et al., 2005). Moreover, Mitchel and Weiss (2014) ruled out attention as an explanation for the difference in learning in the audiovisual aware and audio-only conditions by testing an additional audiovisual condition in which the visual display was uninformative (“audiovisual misinformed”). Participants’ segmentation performance was not significantly different in the audio-only and audiovisual misinformed conditions, suggesting that the significantly greater segmentation in the audiovisual aware condition (identical to the one used in the present study) was not merely a function of increased attention to a facial display. Thus, although we did not directly measure participants’ attention with a cover task, as has been used in some visual statistical learning studies (Bertels et al., 2012), it is unlikely that the difference in segmentation performance between audio-only and audiovisual conditions can be solely attributed to a discrepancy in attention.

Future studies may be able to reduce methodological constraints impacting the extent of learning observed in the present study. In order to replicate Mitchel and Weiss (2014), participants were not given explicit instructions during familiarization, other than to watch the screen. Given this, instructing participants to identify word boundaries during familiarization may provide an avenue for further exploration of top–down eye gaze influences. Higher-order cues in the form of explicit instructions could increase top–down influences, and may allow participants to ignore distracting stimuli and focus their eye gazes on relevant cues (Chen and Zelinsky, 2006). In turn, this could increase the effect sizes seen in this study, with even greater total gaze duration on the facial features relevant to speech segmentation. However, studies such as Filoteo et al. (2010), Nemeth et al. (2013), and Virag et al. (2015) have demonstrated a negative relationship between executive control and implicit learning abilities – those with decreased executive functioning outperform those with greater executive functioning on implicit sequence learning tasks. Since it is believed that speech segmentation relies on implicit learning mechanisms, introducing task instructions may counterintuitively result in a decrease in segmentation performance while increasing eye gaze control. Additionally, in the current study, head movement was minimized during recording of the visual component of the familiarization video. Future studies might examine whether increasing the allowable amount of motion would increase the prominence of segmentation cues and lead to increased segmentation performance.

Finally, the current study provides a foundation for future work in examining the role of specific facial features in visual speech segmentation. Although the current study suggests a relative importance of the mouth, the nature of eye gaze patterns and the task itself provides a significant barrier to isolating the relationship between segmentation performance and an individual facial feature. Over the course of the familiarization video, participants will view nearly all features of the face, and will contend with competing visual interests. By blurring or occluding features, however, studies could examine the contributions of individual features to segmentation performance and determine which visual cues are most critical for accurate segmentation. Lansing and McConkie (1999) found that removing highly attended regions during a given task decreased task performance. Similarly, manipulation of the visual cues made available to participants in a segmentation task could isolate the relationships between individual facial features and segmentation performance, revealing whether highly attended features are both sufficient and necessary for segmentation.

## Author Contributions

LL and AM designed the study, wrote and edited the manuscript, and created the figures. AM created familiarization and segmentation test materials, and completed the analyses. LL coordinated the study and collected the data. This study was conducted as part of LL’s undergraduate honors thesis, under the supervision of AM.

## Conflict of Interest Statement

The authors declare that the research was conducted in the absence of any commercial or financial relationships that could be construed as a potential conflict of interest.
